# Functional Characterization of a Nudix Hydrolase *AtNUDX8* upon Pathogen Attack Indicates a Positive Role in Plant Immune Responses

**DOI:** 10.1371/journal.pone.0114119

**Published:** 2014-12-01

**Authors:** Jose Pedro Fonseca, Xinnian Dong

**Affiliations:** 1 Department of Biology, Duke University, Durham, North Carolina, United States of America; 2 Departamento de Genética, Universidade Federal do Rio de Janeiro, Rio de Janeiro, RJ, Brazil; 3 Howard Hughes Medical Institute–Gordon and Betty Moore Foundation, Department of Biology, Duke University, Durham, North Carolina, United States of America; Texas A&M University, United States of America

## Abstract

Nudix hydrolases comprise a large gene family of twenty nine members in *Arabidopsis*, each containing a conserved motif capable of hydrolyzing specific substrates like ADP-glucose and NADH. Until now only two members of this family, *AtNUDX6* and *AtNUDX7,* have been shown to be involved in plant immunity. *RPP4* is a resistance gene from a multigene family that confers resistance to downy mildew. A time course expression profiling after *Hyaloperonospora arabidopsidis* inoculation in both wild-type (WT) and the *rpp4* mutant was carried out to identify differentially expressed genes in RPP4-mediated resistance. *AtNUDX8* was one of several differentially expressed, downregulated genes identified. A T-DNA knockout mutant (*KO-nudx8*) was obtained from a Salk T-DNA insertion collection, which exhibited abolished *AtNUDX8* expression. The *KO-nudx8* mutant was infected separately from the oomycete pathogen *Hpa* and the bacterial pathogen *Pseudomonas syringae* pv. *maculicola* ES4326. The mutant displayed a significantly enhanced disease susceptibility to both pathogens when compared with the WT control. We observed a small, stunted phenotype for *KO-nudx8* mutant plants when grown over a 12/12 hour photoperiod but not over a 16/8 hour photoperiod. *AtNUDX8* expression peaked at 8 hours after the lights were turned on and this expression was significantly repressed four-fold by salicylic acid (SA). The expression of three pathogen-responsive thioredoxins (*TRX-h2*, *TRX-h3* and *TRX-h5*) were downregulated at specific time points in the *KO-nudx8* mutant when compared with the WT. Furthermore, *KO-nudx8* plants like the *npr1* mutant, displayed SA hypersensitivity. Expression of a key SA biosynthetic gene *ICS1* was repressed at specific time points in the *KO-nudx8* mutant suggesting that *AtNUDX8* is involved in SA signaling in plants. Similarly, *NPR1* and *PR1* transcript levels were also downregulated at specific time points in the *KO-nudx8* mutant. This study shows that *AtNUDX8* is involved in plant immunity as a positive regulator of defense in *Arabidopsis*.

## Introduction

Plants are sessile organisms that evolved remarkable signaling pathways in order to cope with several abiotic and biotic stresses such as pathogen attack. In one branch of the plant immune system there are nucleotide binding leucine-rich repeat (NB-LRR) proteins in the cell that recognize a plethora of pathogen effectors from several kingdoms and activate a cascade of signaling pathways ultimately leading to effector triggered immunity (ETI) [1], [2]. In *Arabidopsis* Columbia ecotype (Col-0), the resistance (R) gene *RPP4* confers resistance to the oomycete pathogen *Hyaloperonospora arabidopsidis* (*Hpa*) isolates Emwa1 and Emoy2 (downy mildew), which involves multiple defense signaling components, including the NPR1 protein among others [3].

Some plant immune responses are associated with NPR1 protein conformational changes induced by redox levels [4]. NPR1 is a well-known master regulator of pathogenesis related (PR) gene expression and salicylic acid (SA) signaling [5], [6]. NPR1 protein resides in the cytoplasm as an oligomer maintained by disulphide bonds that are sensitive to redox changes [4]. Reduction of disulphide bonds cause NPR1 monomer migration to the nucleus and activation of *PR* gene expression. NPR1 also works upstream of SA suppressing expression of *ICS1* and inhibiting SA biosynthesis in a negative feedback loop [7].

Thioredoxins (TRXs) are small cytosolic proteins that act as disulphide reductase proteins [8], [9]. In *Arabidopsis,* there are eight cytosolic types of TRXs, three of which have been related to pathogen attack: TRX-h2, TRX-h3 and TRX-h5 [4]. Of these, *TRX-h3* is the only one constitutively expressed. Reduction of the NPR1 oligomer-to-monomer reaction is catalyzed by cytosolic *TRXs* by the reduction of their intermolecular disulphide bonds. Incubation of NPR1-GFP protein extracts with recombinant TRX-h5 protein can increase the amount of NPR1-GFP monomer [4].

The Nudix (nucleoside diphosphates linked to some moiety X) gene family comprises 29 homologs in *Arabidopsis* and is well conserved across several species and all domains of life (Eukaryotes, Prokaryotes and Archaea) [10]. Its members contain a conserved Nudix box motif “GX5EX7REVXEEXGU” that catalyzes the hydrolytic breakdown of nucleoside diphosphates linked to other moieties by cleavage of chemical bonds. Nudix hydrolases have been shown to catalyze the hydrolysis of nucleoside diphosphates such as nucleotide sugars (ADP-glucose) [11], [12], [13] and pyridine nucleotides such as NADH, NADPH and 8-oxo-GTP [13], [14], [15]. Nucleoside diphosphates are key metabolic intermediates and signaling molecules that are often toxic to the cell. It has been proposed that Nudix hydrolases may have a role as house cleaning enzymes by getting rid of toxic, excessive nucleoside diphosphate and hence maintaining normal cellular homeostasis [12], [16], [17].

Previous phylogenetic analysis of the Nudix gene family in *Arabidopsis* has further divided these into four subfamilies. *AtNUDX8* appears in the fibroblast growth factor type Nudix enzyme (FGFTNE) subfamily in a monophyletic clade. All members in this clade have hydrolase activity towards ADP-ribose and NADH, which is important for defense responses in plants [18]. Most Nudix family members are mainly present in the cytosol (*AtNUDX1* to 11 and 25) but there are also organelle-type Nudix hydrolases which localize to the chloroplast and mitochondrion (AtNUDX14, 15, 19, 23, 26 and 27) in *Arabidopsis* [12]. Enzyme activity has been tested in all Nudix hydrolases (*AtNUDX1*-*AtNUDX27*) in *Arabidopsis*. Only four genes (*AtNUDX2*, *AtNUDX6*, *AtNUDX7* and *AtNUDX10*) showed pyrophosphohydrolase activity towards both ADP-rib and NADH *in vitro* [11]. The involvement of reactive oxygen species (ROS) such as H_2_O_2_ in plant defense against bacterial and fungal pathogens was previously demonstrated [19]. Intensive ROS production from NADH pools during oxidative burst might be required by several defense responses such as lignin formation, antimicrobial action and hypersensitive response during systemic acquired resistance [19]. Nudix enzymes may also play a significant regulatory role in photosynthesis in the reduction of O_2_ to O_2_^+^, which is accompanied by H_2_O_2_ and OH production [17]. Indeed, mutations in house cleaning enzymes and oxidation protective enzymes could generate an imbalance in cellular homeostasis affecting several outputs in plant signaling such as pathogen defense and hormone signaling [20].

Other studies have shown that both *AtNUDX6* and *AtNUDX7* genes are involved in plant defense. *AtNUDX6* is directly involved in the plant immune response as a positive regulator of NPR1-mediated defense [21]. *KO-nudx6* plants showed suppressed levels of several SA-induced, NPR1-dependent genes and also *TRX-h5* involved in SA-induced NPR1 activation. *KO-nudx6* mutant also displays increased NADH levels [21]. Additionally *AtNUDX6* was shown to be involved in SA signaling and its transcript levels induced by SA [21]. *AtNUDX7* on the other hand is involved in plant immunity as a negative regulator of defense response. A previously studied T-DNA knockout line *Atnudt7-1* exhibited an array of pleiotropic effects such as increased resistance to bacterial pathogen *Pseudomonas syringae* pv. tomato (*Pst*) DC3000 (*avrRpt2*) [22] and oomycete pathogen *Hyaloperonospora arabidopsidis* [23], microscopic cell death and constitutive expression of PR genes [14]. *Atnudt7-1* and *Atnudt7-2* knockout lines displayed a small stunted phenotype influenced by edaphic factors and high NADH and ADP-ribose levels [14]. *AtNUDX7* transcript levels are SA insensitive unlike its SA-induced homolog *AtNUDX6* but its transcript was shown to be induced upon *Pst* infection [22].

In this study, we investigated the functional role of the *AtNUDX8* gene (AT5G47240), a previously uncharacterized member of the Nudix hydrolase family, in defense response upon pathogen attack in *Arabidopsis*. In order to perform this study, we first identified an *Arabidopsis* T-DNA insertion knockout mutant for *AtNUDX8* (*KO-nudx8*) and studied disease phenotypes in response to pathogen infection and transcriptional changes observed in pathogen responsive *TRXs* and NPR1*-*dependent genes by quantitative real-time PCR. The results obtained in this study indicate that the *AtNUDX8* gene is a novel element in plant immune responses against pathogen attack.

## Materials and Methods

### Plant Material

Seeds of WT (Col-0) and *KO-nudx8* mutant from Salk T-DNA insertion collection (SALK_092325.55.50.x) were sown in pots containing Metro-Mix 360 soil for 3–4 weeks in a growth chamber. Plants were maintained at 22°C constant temperature and 50% relative humidity. Plants were grown in long day light conditions (16/8 hours of light/dark) for most experiments and a 12/12 hour photoperiod for the small stunted phenotype. For time course, quantitative real-time PCR (qRT-PCR) analysis, three to four leaves from at least three individual plants were collected from the time the lights were turned on in the growth chambers (Zeitgeber time, 0 hour) and five time points were collected (0, 4, 8, 16 and 24 hours). The samples were immediately frozen in liquid nitrogen and stored at −80°C for RNA extraction.

### Molecular analysis of insertion lines

We used a PCR approach to confirm the T-DNA insertion in the *AtNUDX8* (AT5G47240) gene using T-DNA left border LBb1 primer (5′-GCGTGGACCGCTTGCTGCAACT-3′) and gene specific primers LP (5′-GCAATATCTTCGAGCAGCAAC-3′) and RP (5′-TGTTACATTTACCTTTGCGGC-3′). Positive, homozygous T-DNA insertion lines for *AtNUDX8* were allowed to grow for seed collection.

### *Pseudomonas* infection assay

Whole leaves of three-week-old plants were infiltrated with a bacterial suspension (OD_600_ = 0.0002) of *Pseudomonas syringae* pv. *maculicola* (*Psm*) ES4326 in 10 mM of MgSO_4_. We collected infected tissues three days after the infection. Leaf-disks of 28 mm diameter were collected per plant from 8–12 plants. Then, leaf-discs were placed in tubes containing metal beads and 500 µL of MgSO_4_ and ground using Geno-Grinder (SPEX). Suspensions of 20 µL were transferred to a plate containing 180 µL of MgSO_4_ and 10× serial dilutions were made. Aliquots of 10 µL for each serial dilution were then plated on KB medium with antibiotics and incubated at 30°C for 2–3 days after which colony forming units were counted. Statistical analysis were performed using the Student's t test as described previously [24].

### *Hpa* Emwa1 infection assay

*Arabidopsis* seedlings were grown for 10 days at 18°C and 80–100% relative humidity before *Hpa* infection at dawn (0 hour) of growth chamber used. Ten-day-old *Arabidopsis* plants were spray-inoculated (5×10^5^ spores per mL) with an asexual spore suspension of *Hpa*. Disease phenotypes were scored seven days post-infiltration (dpi) using trypan blue staining. A total of 60 infected cotyledons per genotype were used for sporangiophore (SPP) counting and statistical analysis were performed as described previously [25].

### Time course SA treatment

Three-week-old plants were sprayed with 1 mM of salicylic acid and their leaves were collected at five different time points: when lights were turned on (0 hour), 4, 8, 16 and 24 hours. The leaves were immediately frozen in liquid nitrogen and stored at −80°C for subsequent RNA extraction by Trizol method and cDNA synthesis followed by qRT-PCR time course analysis. Mock-treated control plants were sprayed with water and collected at the same time points as SA-treated ones.

### RNA extraction and quantitative real-time PCR (qRT-PCR)

Total RNA was isolated using Trizol reagent (Ambion), and at least 2 µg of RNA were used for cDNA synthesis using SuperScript III reverse transcriptase (Invitrogen) according to the manufacturer's instructions. Amplicons were amplified from cDNA using gene specific primers (see Table 1), and their relative gene expression values were normalized to the *ACTIN2* gene (invariant control) using the ΔΔC_T_ method [26]. Quantitative real-time PCR (qRT-PCR) was performed in a Mastercycler ep realplex S instrument (Eppendorf) using SYBR Green (Applied Biosystems) to monitor dsDNA synthesis.

**Table 1:** Primers used for qRT-PCR.

Primer nomenclature*	Primer sequence (5′-3′)
nudx8f	TGTGGAGCAACCGATGATAA
nudx8r	CGCAGTACCGATGGCTTAAT
trxh3f	CCGTCTTTGCTGACTTAGCC
trxh3r	GTTGGCATTGCCTGAACTTT
trxh5f	GAATTGCAAGCTGTTGCTCA
trxh5r	CACCGACAACACGATCAATG
trxh2f	TAATGTGACGGCAATGCCTA
trxh2r	TGGCACCAATGATTCTTTCA
npr1f	TGCAATTGCTCTCCAACAGCTTCG
npr1r	GCGGCTAAAGCGCTCTTGAAGAAA
PR1f	CTCATACACTCTGGTGGG
PR1r	TTGGCACATCCGAGTC
ics1f	AGGTACGAGCTTTTGTCCAGA
ics1r	TTGACTTGGTGAACTGCAAA
actin2f	GGCAAGTCATCACGATTGG
actin2r	CAGCTTCCATTCCCACAAAC

*f- forward primer; r- reverse primer.

### NPR1 protein analysis

Total protein extracts were obtained from four-week-old *Arabidopsis* plant leaves either treated with SA (24 hours) or non-treated. Plant leaves were flash-frozen in liquid nitrogen and grinded using Geno-Grinder (SPEX). Cell debris was pelleted by centrifugation at 12,000 rpm for 10 min at 4°C to obtain clear protein extracts. Total protein was extracted using plant extraction buffer (50 mM Tris-HCl, pH 7.5, 150 mM NaCl, 10 mM MgCl_2_, 5 mM EDTA, 0.1% Triton X-100, 0.2% Nonidet P-40, 6 mM beta-mercaptoethanol and 1 protease inhibitor cocktail (Roche) tablet per 100 mL of solution). Protein concentration for the various extracts were measured according to Bradford's method [27]. Aliquots of protein extract were separated on a 12% SDS-PAGE gel [28] and transferred to a nitrocellulose membrane. Western analysis was performed using an antibody against NPR1 protein as described previously [29].

### Data analysis

All the measurements were performed with three biological replicates grown independently. Significant differences between data sets were evaluated using the Student's t test with GraphPad Prism software.

## Results and Discussion

### *KO-nudx8* mutant displays a small, stunted photoperiod-dependent phenotype

In order to identify genes involved in RPP4*-*mediated resistance against *Hpa* Emwa1, a time course gene expression profiling was performed after *Hpa* infiltration (0 hour, 2 days post-infiltration, 4 dpi and 6 dpi) for the *rpp4* mutant and WT. This microarray data was submitted in NCBI's Gene Expression Omnibus database (GEO, accession number GSE22274) [30].

We found 106 differentially expressed genes between WT and *rpp4.* The Athena database (http://www.bioinformatics2.wsu.edu/Athena) was used to analyse the promoter regions to identify regulatory cis-acting elements for all the differentially expressed genes. Remarkably, it was identified that the most prominent cis-element present in the promoters of 30 genes analysed was the evening element (EE). The evening element is known to be regulated by the *CIRCADIAN CLOCK-ASSOCIATED 1* gene (*CCA1*), and the fact the EE can be found in the promoter region of some defense genes suggests a link between the circadian clock and plant defense. It was previously reported that *CCA1* transcription factor can control expression of several *R* genes [25], establishing a link between the circadian clock and plant immunity. We obtained T-DNA insertion mutants of these genes and profiled their disease susceptibility phenotype to *Hpa* infection and *Pseudomonas syringae* pv. *maculicola* (*Psm*) ES4326 followed by functional characterizations of susceptible mutants.

**Figure 1:**
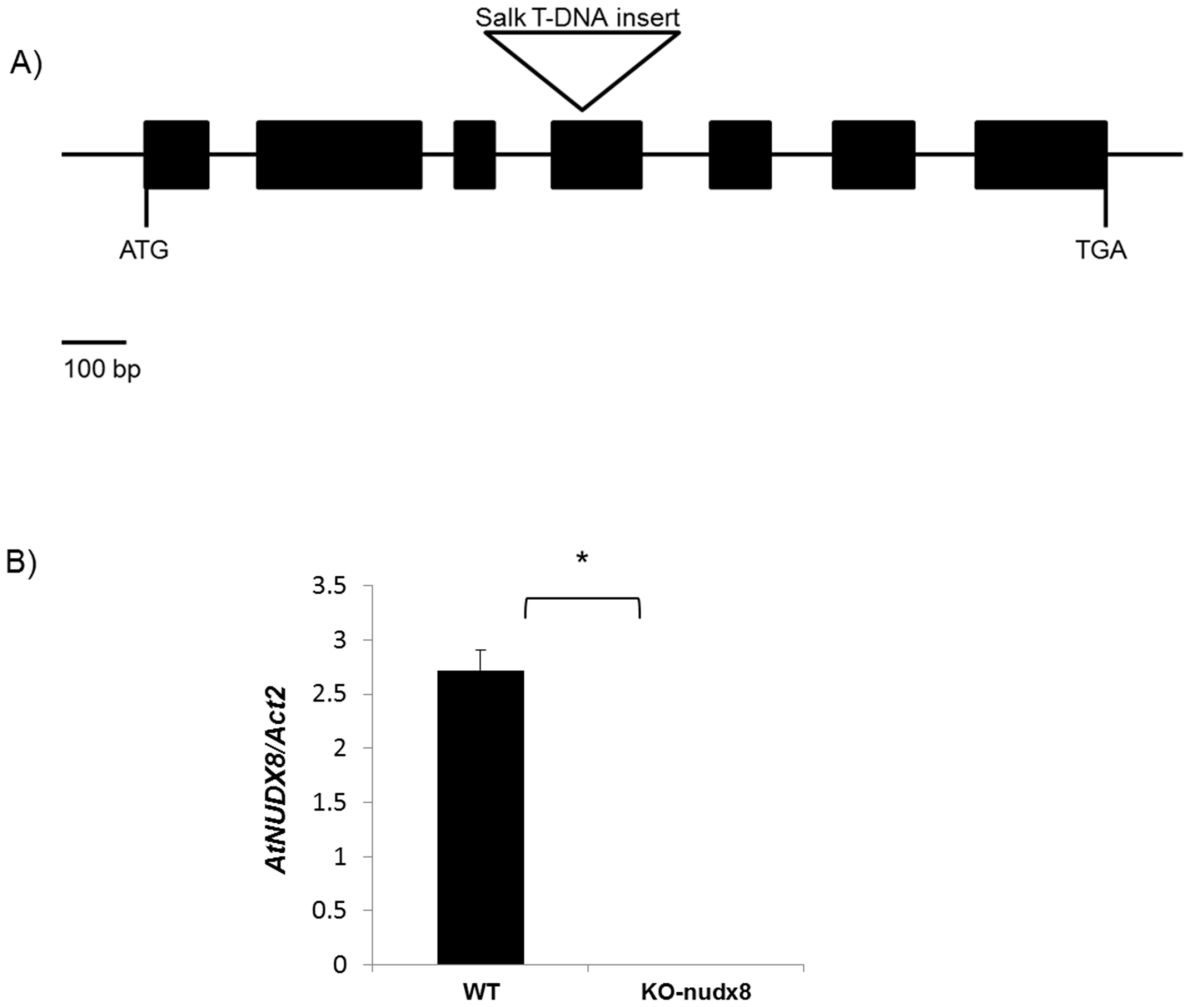
*AtNUDX8* expression is completely abolished in the *KO-nudx8* mutant. A) Diagram showing Salk T-DNA insertion (SALK_092325.55.50.x) position based on the flanking sequence. *AtNUDX8* gene has seven exons (black boxes) and six introns. The T-DNA insertion is located in exon 4 and position +963 relative to the start codon. B) qRT-PCR showing relative expression of *AtNUDX8* in WT and *KO-nudx8* mutant plants where bars represent change in expression of *AtNUDX8* transcript in the WT and knockout mutant plants relative to the internal control *ACTIN2.* The control was selected as a reference gene since it does not vary in the different conditions tested. Data are reported as means ±SD of three independent biological replicates. Asterisk indicates significant difference according to Student's t test (*P*<0.05).

Using PCR we genotyped a Salk T-DNA insertion line (SALK_092325.55.50.x) for the *AtNUDX8* gene (AT5G47240) to confirm its homozygosity. The T-DNA insertion was located in exon 4 and position +963 relative to the start codon (Fig. 1A) based on the sequence obtained from the left-border T-DNA primer of SALK_092325.55.50.x (http://signal.salk.edu/). We performed quantitative real-time PCR to measure the expression of *AtNUDX8* gene in WT and homozygous *KO-nudx8* mutant plants containing the T-DNA insertion, and we demonstrated that the expression of *AtNUDX8* was abolished in the Salk mutant line (Fig. 1B).

**Figure 2:**
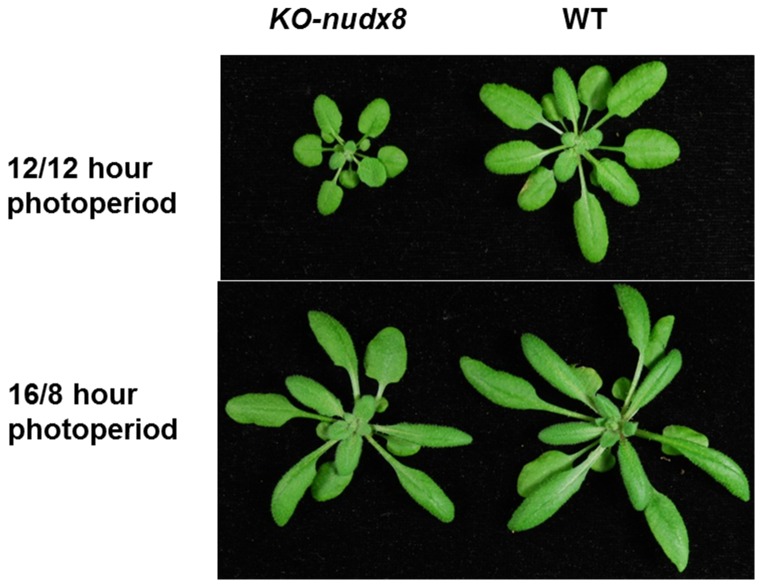
AtNUDX8 displays a small stunted phenotype. Three-week-old WT and *KO-nudx8* plants grown over a 12/12 hour (light/dark) and 16/8 hour photoperiod.

Furthermore, we observed a small stunted phenotype for *KO-nudx8* plants when grown on 12/12 hour photoperiod light (Fig. 2) similar to the *AtNUDX7* gene. The same phenotype was not observed for short or long day light exposures (8/16 hours or 16/8 hours of light/dark).

### *KO-nudx8* plants exhibit a significant increase in susceptibility to pathogen infection

**Figure 3:**
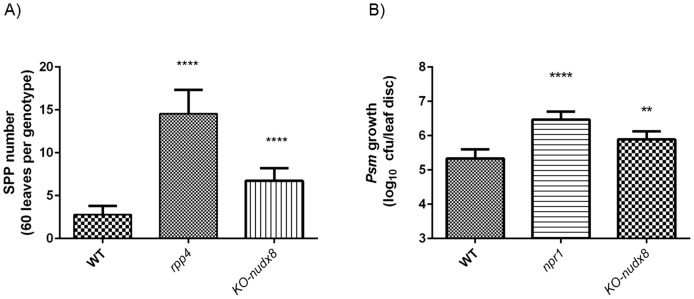
KO-*nudx8* mutant is more susceptible to infection against bacterial and oomycete pathogen than WT plants. A) KO-*nudx8* mutant is more susceptible to *Hyaloperonospora arabidopsidis* Emwa1 infection than the WT (three-fold change). Sporangiophore (SPP) count after *Hp*a Emwa1 infection at dawn. Ten-day-old *Arabidopsis* plants were inoculated (5×10^5^ spores per mL) with asexual spore suspension of *Hpa*. Disease phenotypes were scored seven days post-infiltration (dpi) after trypan blue staining. B) *KO-nudx8* plants are more susceptible to the bacterial pathogen *Psm* ES4326 than WT (0.6 fold change Log_10_). Infected tissue was collected three days after infection. All experiments were carried out with at least three biological replicates. Error bars represent 95% confidence intervals of log-transformed data (n = 7). Asterisks indicates significant difference according to Student's t test (*P*<0.05).

*KO-nudx8* plants were significantly more susceptible to infection with oomycete pathogen *Hpa* Emwa1 in comparison with WT (Fig. 3A). Similarly, *KO-nudx8* also exhibited a significant increase in bacterial growth of *Psm* ES4326 when compared to WT plants (Fig. 3B). Together, these results indicate that *AtNUDX8* is involved in plant defense responses upon pathogen attack whereas plants without a functional copy of *AtNUDX8* exhibit a disease susceptible phenotype. Moreover, *AtNUDX8* transcript levels were found to be downregulated in the *rpp4* mutant compared to WT plants from microarray data upon *Hpa* infection (GEO accession number GSE22274). This result indicates that the *AtNUDX8* gene is involved in RPP4-mediated defense against *Hpa*. In addition to being susceptible to *Hpa,* we also demonstrated increased disease susceptibility in *KO-nudx8 mutant* for *Psm* ES4326 infection, suggesting a common defense mechanism for both types of pathogens analyzed in this study. All these factors indicate that *AtNUDX8* acts as a positive regulator of defense in *Arabidopsis*. Members of the NB-LRR protein class can confer resistance to both *Hpa* and *Psm*. The idea that one gene in plant immunity may confer resistance against more than one pathogen type is not new [31] and it has recently been shown that pathogen effectors interact within a limited set of highly connected cellular hubs in plant immunity [32]. There is growing evidence, including for the gene presented in this study, that some defense genes are involved in plant resistance to more than one pathogen such as *RPP4* and *PgPR10-1* genes amongst others [31], [32].

**Figure 4:**
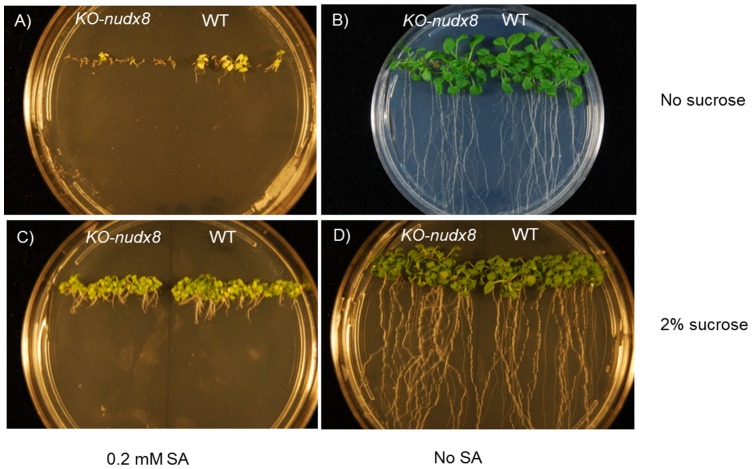
*KO-nudx8* mutant hypersensitivity to SA compared to WT. Seeds were plated on MS medium with 0.2 mM SA and without SA. *KO-nudx8* and WT seedlings were grown for two weeks on A) MS, 0.2 mM SA, no sucrose, B) MS, no sucrose, C) MS, 0.2 mM SA, 2% sucrose and D) MS, 2% sucrose.

Additionally, in a similar way to the *npr1* mutant, *KO-nudx8* exhibited hypersensitivity to SA compared with WT control plants. Two-week-old *KO-nudx8 Arabidopsis* seedlings displayed impaired cotyledon germination in media with 0.2 mM SA compared to the WT control with the same conditions (Fig. 4). Control plants in Murashige and Skoog (MS) medium without SA developed normally. When sucrose was subtracted from MS, the control plants grew normally in MS media without SA, but in 0.2 mM SA lacking sucrose plates there was a marked difference between WT and *KO-nudx8* plants (Fig. 4A and 4B) whereby WT plants germinated cotyledons but *KO-nudx8* barely germinated or developed cotyledons. These results suggest that the *AtNUDX8* gene is involved in SA signaling and also, that in nutrient poor, energy deprived conditions *AtNUDX8* is required for growth and detoxification.

### Effect of disruption of the *AtNUDX8* gene in pathogen induced thioredoxins *TRX-h1*, *TRX-h2* and *TRX-h3*

**Figure 5:**
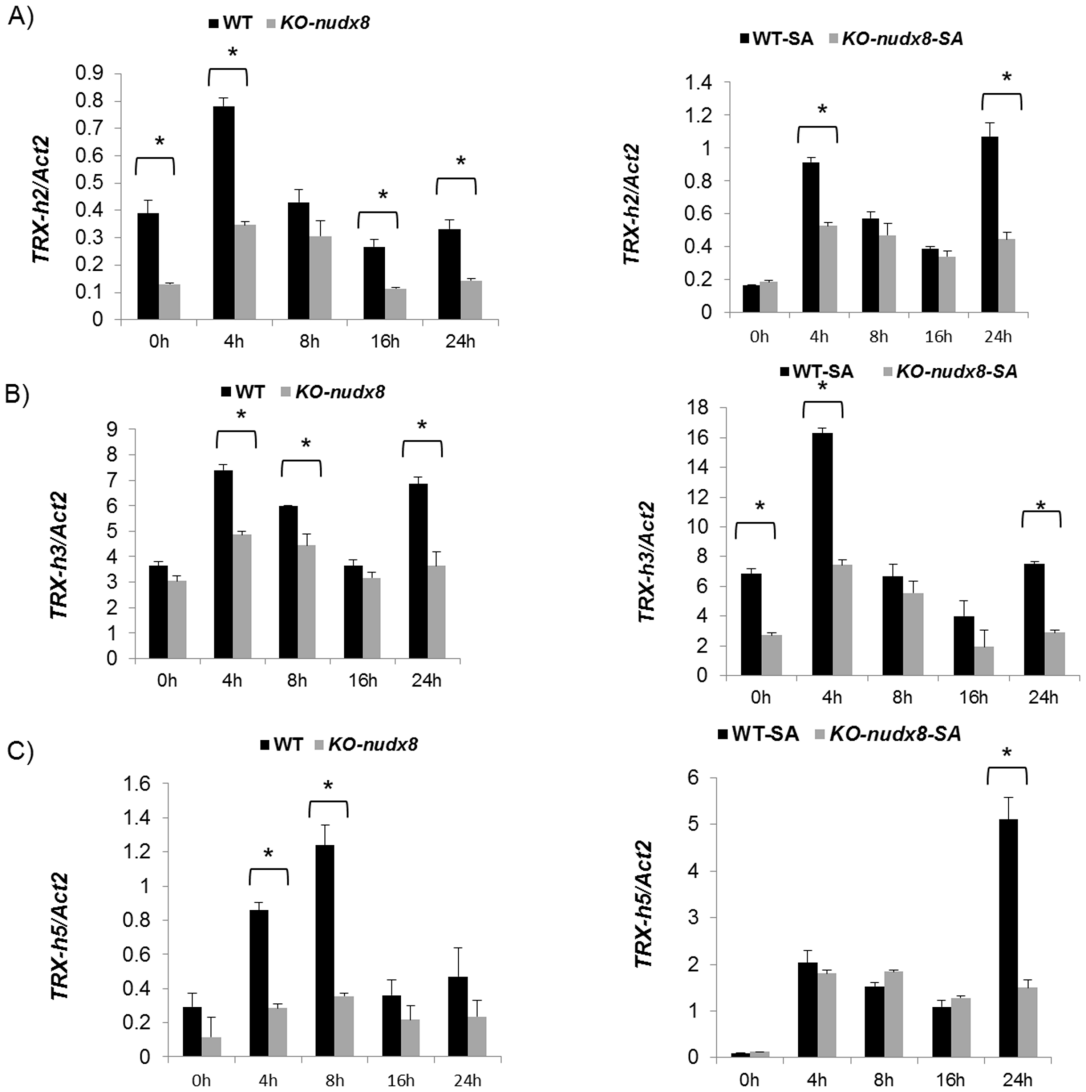
Pathogen responsive thioredoxins *TRX-h2, TRX-h3* and *TRX-h5* are downregulated in the *KO-nudx8* mutant. Time course expression analysis by qRT-PCR of pathogen responsive thioredoxins in A) *TRX-h2,* B) *TRX-h3* and C) *TRX-h5.* Relative expression levels were measured from WT control and *KO-nudx8* mutant with and without 1 mM SA treatment for each gene tested. Three-week-old plants were sprayed with 1 mM of SA and samples were collected at different time points. Bars represent change in relative expression for each thioredoxin in WT and *KO-nudx8* mutant plants (with and without SA) relative to the internal control *ACTIN2* used as a reference gene (since it does not vary under the different conditions and treatments tested). Relative expression values were normalized to *ACTIN2* mRNA using the ΔΔCT method. Data are reported as means ±SD of three independent biological replicates. Asterisks indicate significant differences according to Student's t test (*P*<0.05). Zeitgeber time is indicated from the time the lights were turned on at 0 hour for a period of 24 hours.

NPR1 conformational changes in the cytosol can influence plant immunity. These conformational changes were shown to be modulated by *TRXs* that promote NPR1 monomerization [4]. In order to verify the role of the *AtNUDX8* gene in thioredoxin-dependent NPR1 oligomer-monomer reduction we analyzed the gene expression of three thioredoxins (*TRX-h2*, *TRX-h3* and *TRX-h5*) which are known to be upregulated upon pathogen response. The transcript levels of all three pathogen-regulated thioredoxins were significantly reduced in *KO-nudx8* plants at specific time points in SA-treated and untreated plants (Fig. 5A, 5B and 5C). This result indicates that *AtNUDX8* can modulate expression of the three pathogen induced thioredoxins at specific time points. Further investigation is required to understand how the *AtNUDX8* coding enzyme interacts with thioredoxins to modulate NPR1 conformational changes. AtNUDX8 interaction with thioredoxins could be by direct protein-protein interaction or by changing the levels of a metabolic intermediate by substrate hydrolysis that consequently will affect the redox pools in the cell. AtNUDX8 could act as a potential house-cleaning enzyme in the process of removing excessive reactive oxygen species (ROS) towards the end of a pathogen attack event. Mutations in the *AtNUDX8* gene is likely to cause changes in redox homeostasis in the cell by affecting levels of a key metabolic intermediate impacting several plant physiological processes such as pathogen defense and SA biosynthesis.

### *AtNUDX8* expression peaks at 8 hours and is significantly repressed four-fold by Salicylic Acid

**Figure 6:**
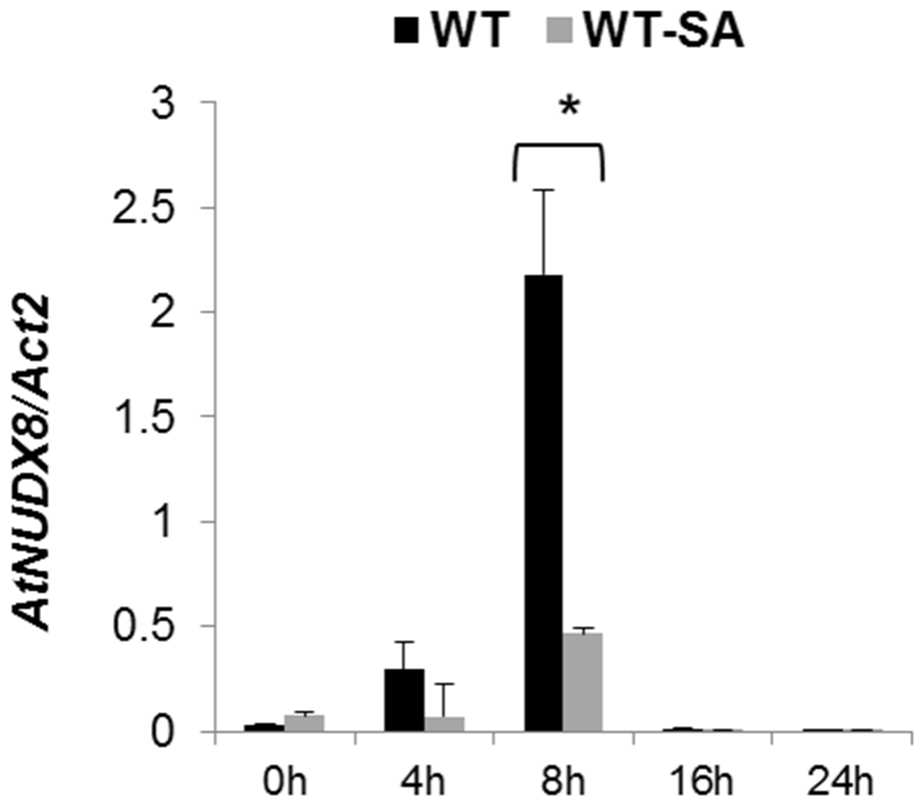
*AtNUDX8* expression peaks at eight hours and is repressed by SA. qRT-PCR analysis of *AtNUDX8* relative expression levels in the leaves of WT plants without SA treatment and 1 mM SA in a 24 hour time course expression analysis. Three-week-old plants were sprayed with 1 mM of SA and water (mock control), and samples were collected at different time points. Bars represent change in expression of *AtNUDX8* transcript in WT plants without SA and SA-treated relative to the internal control *ACTIN2* used as reference gene (since it does not vary under the different conditions and treatments tested). Relative expression values were normalized to *ACTIN2* mRNA using the ΔΔCT method. Data are reported as means ±SD of three independent biological replicates. Asterisk indicates significant difference according to Student's t test (*P*<0.05). Zeitgeber time is indicated from the time the lights were turned on at 0 hour for a period of 24 hours.

We examined *AtNUDX8* transcript levels in a time course expression analysis, and we observed that this gene, unlike other Nudix homologs previously described involved in defense [21], [22], displayed a typical circadian-like pulse gene expression with expression peaking at 8 hours after the lights were turned on (Fig. 6). A time course expression profiling for *AtNUDX8* from Diurnal database (http://diurnal.mocklerlab.org/) profiled similar results with *AtNUDX8* expression also peaking at 8 hours (Zeitgeber time). Although *AtNUDX8* transcript exhibited a rhythmic circadian-like expression, further experiments will be required to validate *AtNUDX8* as a *bona fide* binding site for CCA1 or other circadian clock transcription factors by ChIP qRT-PCR or Yeast-One-hybrid experiments. Additionally, *AtNUDX8* transcription was repressed four-fold at 8 hours in response to 1 mM SA treatment compared with mock-treated plants. Regulation of defense gene expression must be controlled between positive and negative regulators in order to avoid an overactivation of defense in plants, which would lead to impaired normal cell activity and loss of redox homeostasis in the cell.

### Effect of disruption of *AtNUDX8* in NPR1-mediated defense

**Figure 7:**
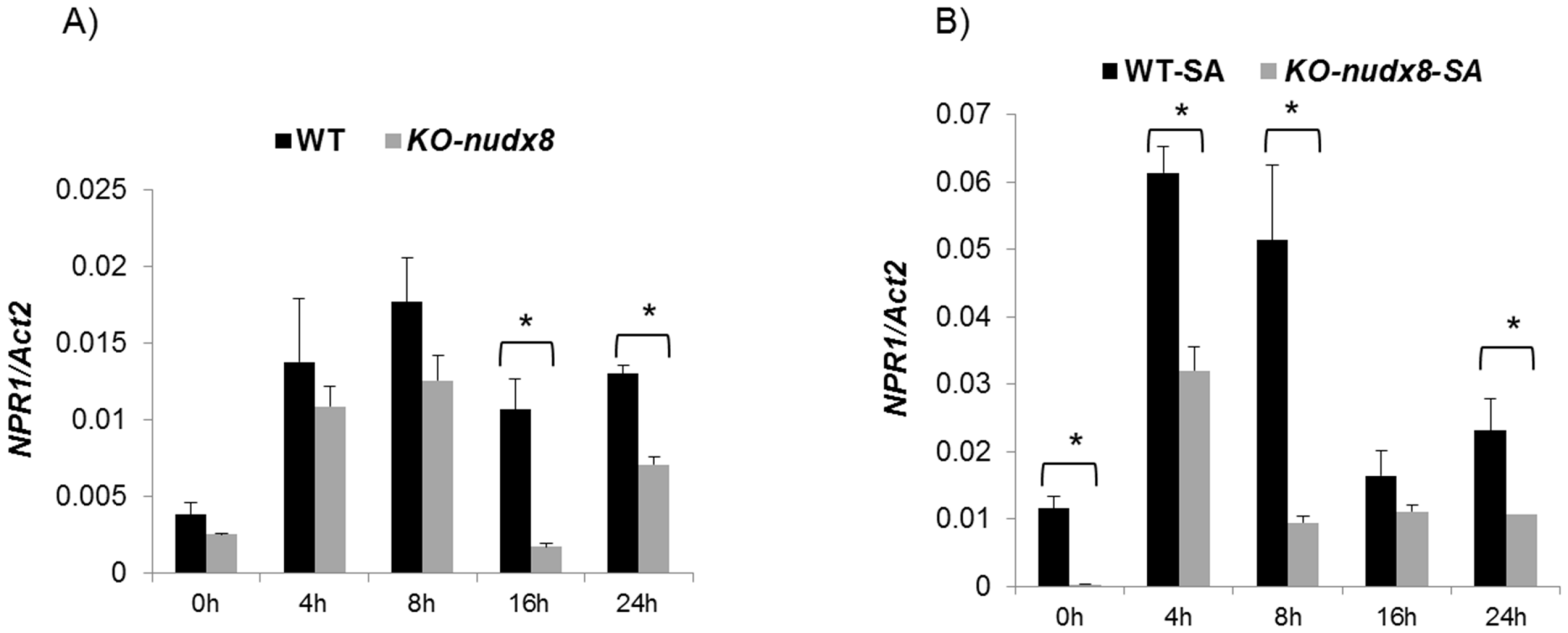
Time course analysis showing *NPR1* gene downregulation in *KO-nudx8* mutant in relation to WT plants. qRT-PCR analysis of *NPR1* relative expression levels in a 24 hour time course in the leaves of WT plants and *KO-nudx8* mutant. A) No SA treatment, B) 1 mM SA treatment. Expression of *NPR1* is downregulated in several time points in the *KO-nudx8* mutant compared to WT. Three-week-old plants were sprayed with 1 mM of SA and samples were collected at different time points. Bars represent change in expression of *NPR1* transcript in the WT and *KO-nudx8* plants relative to the internal control *ACTIN2* used as reference gene (since it does not vary under the different conditions and treatments tested). Relative expression values were normalized to *ACTIN2* mRNA using the ΔΔCT method. Data are reported as means ±SD of three independent biological replicates. Asterisks indicate significant differences according to Student's t test (*P*<0.05). Zeitgeber time is indicated from the time the lights were turned on at 0 hour for a period of 24 hours.

**Figure 8:**
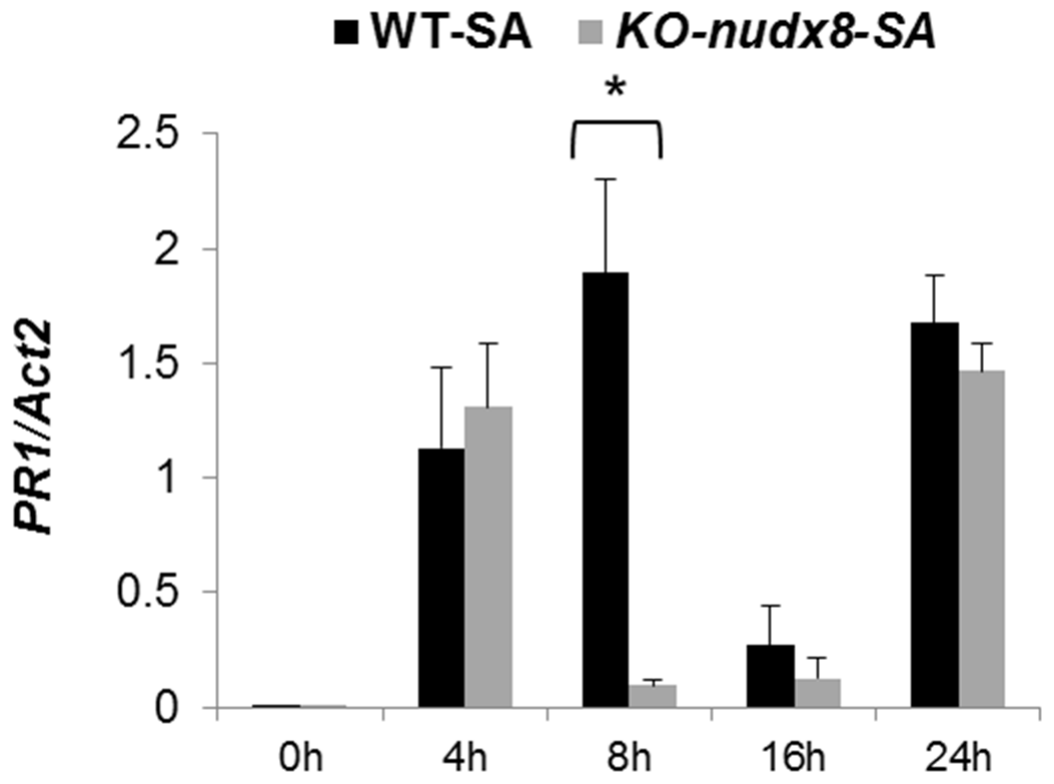
Time course analysis showing *PR1* downregulation at 8 hours in the *KO-nudx8* mutant. qRT-PCR analysis of *PR1* relative expression levels in a 24 hour time course in the leaves of WT plants and *KO-nudx8* mutant treated with 1 mM SA. Expression of *PR1* was downregulated at 8 hours in the *KO-nudx8* mutant compared to WT. Three-week-old plants were sprayed with 1 mM of SA and samples were collected at different time points. Bars represent change in expression of *PR1* transcript in the WT and *KO-nudx8* plants relative to the internal control *ACTIN2* used as reference gene (since it does not vary under the different conditions and treatments tested). Relative expression values were normalized to *ACTIN2* mRNA using the ΔΔCT method. Data are reported as means ±SD of three independent biological replicates. Asterisk indicates significant difference according to Student's t test (*P*<0.05). Zeitgeber time is indicated from the time the lights were turned on at 0 hour for a period of 24 hours.

**Figure 9:**
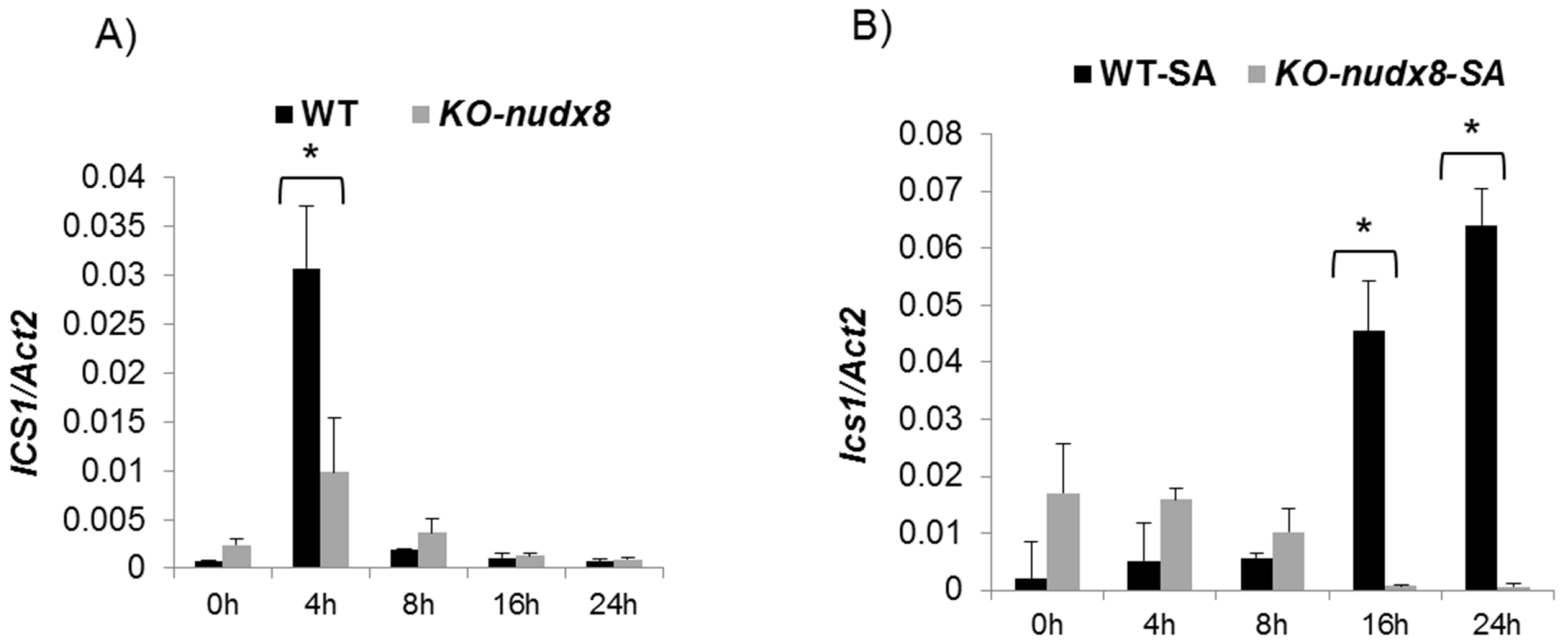
Time course analysis showing *ICS1* gene downregulation in the *KO-nudx8* mutant in relation to WT plants. **A**) No SA treatment, B) 1 mM SA treatment. *ICS1* expression peaks at 4 hours (no SA) and is downregulated in the *KO-nudx8* mutant in specific time points for both SA-untreated and SA-treated plants. Three-week-old plants were sprayed with 1 mM of SA and leaf samples were collected at different time points. Bars represent change in expression of *ICS1* transcript in the WT and *KO-nudx8* plants relative to the internal control *ACTIN2* used as reference gene (since it does not vary under the different conditions and treatments tested). Relative expression values were normalized to *ACTIN2* mRNA using the ΔΔCT method. Data are reported as means ±SD of three independent biological replicates. Asterisk indicates significant difference according to Student's t test (*P*<0.05). Zeitgeber time is indicated from the time the lights were turned on at 0 hour for a period of 24 hours.

To study the role of *AtNUDX8* in NPR1-mediated defense and the SA signaling pathway, we performed a time course expression analysis to measure the transcript levels of *NPR1, PR1* and the SA biosynthetic gene *ICS1* in *KO-nudx8* and WT plants. *NPR1* transcript levels were significantly downregulated in *KO-nudx8* plants at specific time points in SA-induced plants and untreated (no SA) (Fig. 7). *PR1* gene expression was also reduced significantly at 8 hours in the *KO-nudx8* mutant compared to WT in SA-induced plants (Fig. 8). Similarly, expression of the *ICS1* gene was significantly repressed at 4 hours in untreated plants and at 16 and 24 hours in SA-treated plants in the *KO-nudx8* mutant plants compared with the WT (Fig. 9). This downregulation of *ICS1* suggests that *AtNUDX8* is involved in the SA signaling pathway by indirectly acting on *ICS1* transcript levels. SA is synthesized from chorismate through isochorismate by the action of *ICS1*. The fact that *AtNUDX8* acts to modulate, not only thioredoxin-dependent NPR1 oligomer-monomer formation, but also *ICS1* transcript levels, indicates that the *AtNUDX8* gene can impact SA levels in the plant indirectly since NPR1 has been shown to maintain SA levels. NPR1 acts not only downstream to SA but also upstream as a negative regulator of *ICS1* gene expression, inhibiting SA [7], [33].

**Figure 10:**
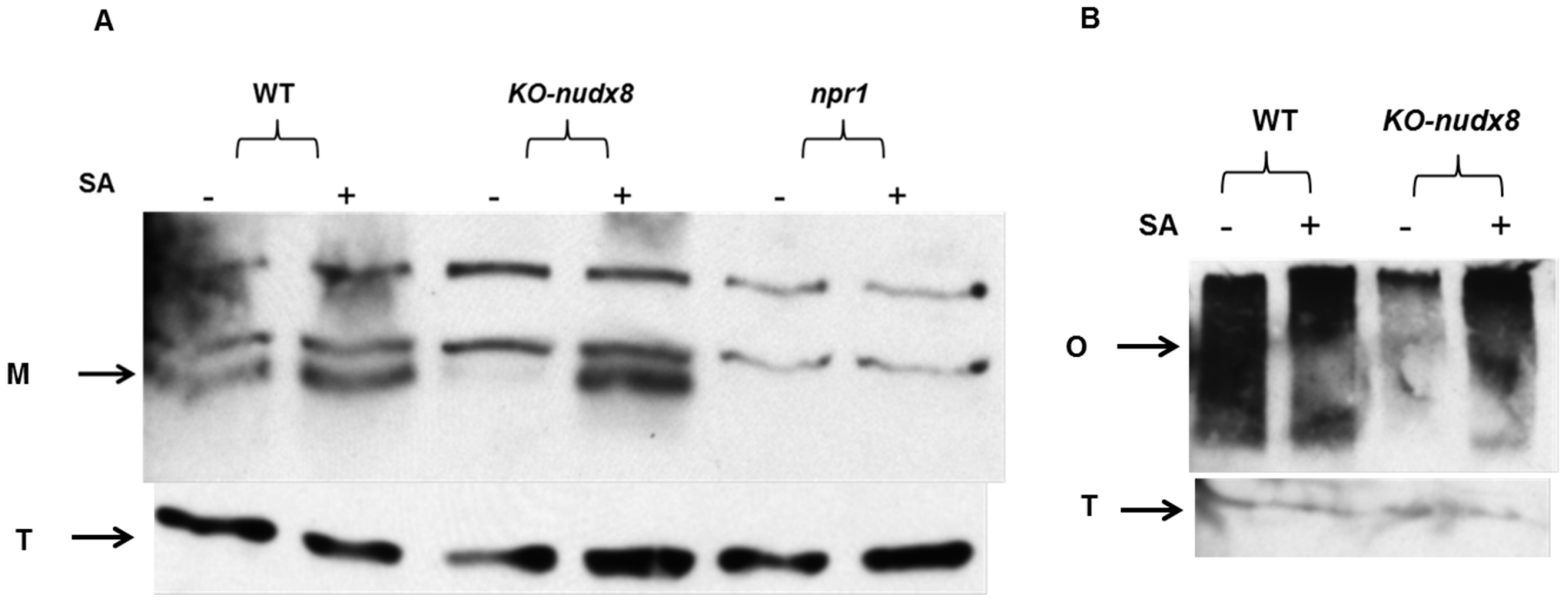
NPR1 protein detection in protein extracts from WT and *KO-nudx8* mutant plants. Total protein was extracted from WT, *KO-nudx8* and *npr1* mutant plants with protein extraction buffer. Leaf samples were collected for each genotype from 1 mM SA treated (+) and water treated (−) samples. Proteins were analyzed by reducing and non-reducing SDS-PAGE and western blotting using anti-NPR1 (A and B respectively). Western blot shows NPR1 monomer protein detection (M) and NPR1 oligomer (O). NPR1 is absent in the *KO-nudx8* mutant without SA treatment grown in long day conditions. Total protein loading control (T) for each sample is shown at the bottom. Zeitgeber time is indicated from the time the lights were turned on at 0 hour for a period of 24 hours.

Furthermore, we did not detect NPR1 protein in the *KO-nudx8* mutant without SA treatment (Fig. 10A), but we were able to detect with SA treatment. The fact that NPR1 protein was detected in the SA-treated *KO-nudx8* mutant indicates that other pathways and genes can compensate for *AtNUDX8* loss in SA-dependent NPR1 signaling. A similar result was observed for NPR1 in non-reducing conditions with a marked decrease in NPR1 oligomer for the *KO-nudx8* mutant compared to the WT plants without SA treatment (Fig. 10B). No difference in oligomer level was observed upon SA treatment.

A previous study [21] demonstrated a role for an *AtNUDX8* paralog, *AtNUDX6*, in NPR1-mediated defense and SA biosynthesis. Interestingly *AtNUDX6* belongs to the same Nudix subfamily as *AtNUDX8*, grouping together in a monophyletic clade [18]. It has been shown that both *AtNUDX6* and *AtNUDX7* genes are positive and negative regulators of defense response respectively [21], [22]. Our findings indicate a positive role for the *AtNUDX8* gene in NPR1-mediated defense in *Arabidopsis* in a similar way to *AtNUDX6,* both antagonizing *AtNUDX7*'s negative role in defense. Indeed, both *AtNUDX8* and *AtNUDX6* genes may act synergistically to promote plant immunity through the regulation of NPR1 conformation in the cytosol albeit by slightly different mechanisms. Contrarily to *AtNUDX6*, which is induced upon SA treatment, *AtNUDX8* was repressed up to four-fold by SA when its expression was at its peak at 8 hours (see Fig. 5), indicating slightly different regulatory functions for these family member homologs upon SA signaling. A fine-tuning of both positive and negative regulation of defense related genes is required in order to avoid excessive activation of defense reaction upon pathogen infection.

**Figure 11:**
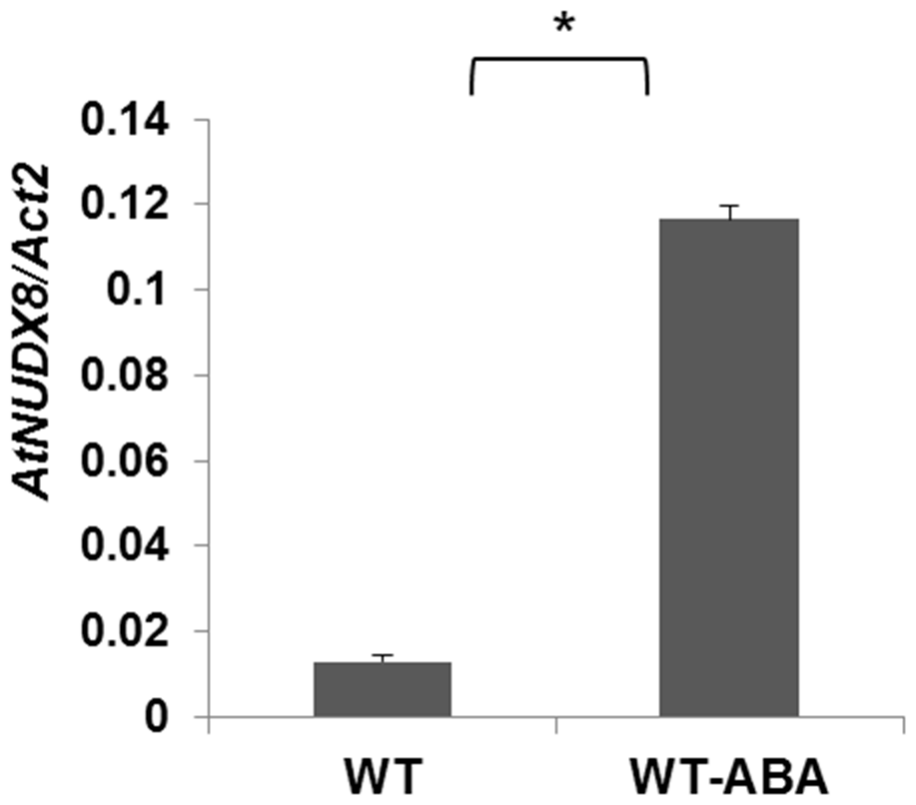
*AtNUDX8* transcript levels are induced by ABA. qRT-PCR analysis of *AtNUDX8* relative expression levels in the leaves of WT plants treated with 50 µM of ABA (WT-ABA) and untreated (WT). Three-week-old plants were sprayed with 50 µM ABA and samples were collected at different time points. Bars represent change in expression of *AtNUDX8* transcript in the untreated (WT) and ABA-treated plants (24 hours) relative to the internal control *ACTIN2* used as reference gene (since it does not vary under the different conditions and treatments tested). Relative expression values were normalized to *ACTIN2* mRNA using the ΔΔCT method. Data are reported as means ±SD of three independent biological replicates. Asterisk indicates significant difference according to Student's t test (*P*<0.05).

Furthermore, we demonstrated in this study that *AtNUDX8* transcript was significantly induced by ABA treatment (Fig. 11) indicating a possible functional role in abiotic stress responses such as osmotic stress, drought and salinity. Further investigations will be required to assess the involvement of the *AtNUDX8* gene in such events. Microarray data from the eFP *Arabidopsis* browser (http://bar.utoronto.ca/efp/cgi-bin/efpWeb.cgi) corroborates this hypothesis as it shows that *AtNUDX8* transcription is upregulated under several abiotic stress parameters such as osmotic stress, salt, drought, genotoxic and wounding.

## Conclusions

This work lays the foundation for the study of a novel, previously uncharacterized member of the Nudix hydrolases, *AtNUDX8* gene, in plant immunity and SA signaling in *Arabidopsis*. Plants without a functional copy of the *AtNUDX8* gene were significantly more susceptible to infection than WT plants against *Hpa* and *Psm* ES4326 pathogen infection, indicating a positive role in plant defense for the *AtNUDX8* gene. Additionally, *KO-nudx8* plants displayed a small, stunted phenotype when grown over a 12/12 hour photoperiod (light/dark) but not over a 16/8 hour photoperiod. *KO-nudx8* plants also displayed a phenotype of SA hypersensitivity compared to the WT control when grown in SA-containing media. All the three pathogen-responsive *TRXs* (*TRX-h2*, *TRX-h3* and *TRX-h5*) were downregulated at specific time points in the *KO-nudx8* mutant indicating that *AtNUDX8* may have an important function in the oligermerization state of NPR1 indirectly by the modulation of pathogen responsive *TRXs*. We also demonstrated that *KO-nudx8* mutant displayed significantly lower *NPR1, PR1* and *ICS1* transcription levels at specific time points compared with the WT indicating that *AtNUDX8* plays a role in NPR1-dependent defense pathway and SA signaling. Collectively, these results demonstrate that the *AtNUDX8* gene is involved in RPP4-mediated resistance by the modulation of pathogen responsive *TRXs* that, in turn, could cause changes in NPR1 reduction state. The precise mechanism of how *AtNUDX8* modulates *TRXs* remains unknown. We speculate that *AtNUDX8* is an important element linking redox or energy metabolism and biotic stress signaling by affecting levels of a yet unknown metabolic intermediate by substrate hydrolysis.

*AtNUDX8* exhibited a typical circadian-like rhythmic expression peaking at 8 hours after the lights were turned on indicating a possible involvement of *AtNUDX8* in light regulation or the circadian clock but further experiments will be necessary to determine *AtNUDX8* as a *bona fide* target for core clock transcription factors such as *CCA1* transcription factor. Indeed, out of the three Nudix members shown to be involved in plant immune responses thus far (*AtNUDX8, AtNUDX6* and *AtNUDX7*), only *AtNUDX8* has EE binding motifs on its promoter region.

The emerging picture indicates an active role for Nudix hydrolases in plant immunity and future work will be necessary to clarify our understanding of, not only *AtNUDX8* gene, but also other Nudix family members in biotic and also abiotic stress such as drought, osmotic stress and wounding amongst others. These results argue for potential biotechnological applications of the *AtNUDX8* gene in commercial plant systems for pathogen resistance and possibly abiotic stress tolerance, although further work will be required to assess the functional role of *AtNUDX8* in abiotic stress responses. Future experiments such as enzyme activity assays will be important to know which substrate the AtNUDX8 enzyme affects. Global expression profile studies between *KO-nudx8* mutant and WT will help to identify differentially expressed genes and pathways regulated by *AtNUDX8.* Additionally, differences in metabolite signatures detected by mass spectrometry between *KO-nudx8* and WT plants will be useful in helping to identify which cell metabolites are affected by the AtNUDX8 enzyme.
